# Class of gauge-invariant models of quantum electrodynamics with nonlocal interaction

**DOI:** 10.1038/srep45386

**Published:** 2017-04-03

**Authors:** Tao Mei

**Affiliations:** 1 Department of Journal, Central China Normal University, Wuhan, Hubei PRO, People’s Republic of China

## Abstract

We present a class of gauge-invariant models of quantum electrodynamics with nonlocal interaction. The models have translation, Lorentz and gauge invariance and reduce to the conventional local quantum electrodynamics under the appropriate limit conditions, both the equations of motion of the charged particle and electromagnetic field obtained by the action principle lead to the normal form of current conservation. Quantization of the models is realized by taking advantage of the formalism based on the Yang-Feldman equations and the Lehmann-Symanzik-Zimmermann reduction formulas. Finally, we employ a special choice of the models to calculate the vacuum polarization as an example to demonstrate the possibility of establishing a theory of quantum electrodynamics without divergence.

There is a large literature on nonlocal field theory (for a review and references, see refs 1, 2). One of the motivations for investigating nonlocal field theory is to establish a theory without divergence; since the renormalized theory of local gauge fields leads to finite predictions and to an unprecedented agreement between theory and experiment, if divergences still appear in the corresponding nonlocal theory and must still be eliminated by a renormalization procedure, then such a nonlocal field theory is not interesting.

Of course, according to the contemporary point of view, any field theory must be renormalized; if a nonlocal field theory involves finite renormalization constants, then such a theory accords with the contemporary point of view and standard mathematical theory at the same time, since standard mathematical theory allows ignoring small or finite quantities (but ignoring an infinitely large quantity does not accord with standard mathematical theory)^3^.

Some models of quantum electrodynamics (QED) with nonlocal interaction have been proposed^4^,^5^,^6^. In this paper, we present a class of models of QED with nonlocal interaction.

After looking back on some known nonlocal models of QED, we present the action of a class of models and the equations of motion of a charged particle and electromagnetic field obtained by the action principle. Translation, Lorentz and gauge invariance of the models are proved, and we prove that both the equations of motion of a charged particle and electromagnetic field lead to the normal form of current conservation. The models are reduced to the conventional local QED under the appropriate limit condition.

Similar to some known nonlocal modes of QED, for guaranteeing the gauge invariance of the theory, the form of the models presented in this paper is far from that of conventional field theory.

Next, based on the fact that a free charged particle and free electromagnetic field still obey the local Dirac equation and the local Maxwell equation of free fields, respectively, quantization of the model is realized by taking advantage of the formalism based on the Yang-Feldman equations and the Lehmann-Symanzik-Zimmermann reduction formulas. Some properties, e.g., Lorentz and gauge invariance, unitarity and causality of the elements of the *S*-matrix, are discussed.

The models presented in this paper provide a wide range of choices; we employ a special choice of the models to calculate the vacuum polarization as an example to show the possibility of establishing a theory of QED without divergence.

All symbols and conventions of this paper follow ref. 7, for example, *g*^*αβ*^ = *g*_*αβ*_ = diag (+1, −1, −1, −1) and *A* · *B* = *A*^*λ*^*B*_*λ*_ = *A*^0^*B*^0^ − ***A*** · ***B***.

## Looking back on models of quantum electrodynamics with nonlocal interaction

As is well known, the action of the conventional local QED is


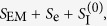


where


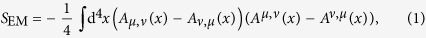



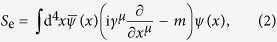



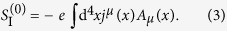


In (3), 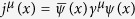. The theory is invariant under the gauge transformation


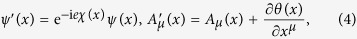


where *θ*(*x*) is an arbitrary scalar function. The relation between the two functions *χ*(*x*) and *θ*(*x*) in (4) reads


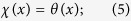


and both the equations of motion of the charged particle and electromagnetic field obtained by the action principle lead to the current-conservation equation


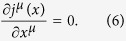


Since 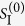 given by (3) can be written in the form 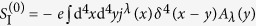, by replacing *δ*^4^(*x* − *y*) by a scalar function *f*(*x* − *y*), which is independent of *ψ*(*x*) and *A*^*μ*^(*x*), 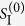 becomes


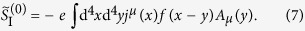


Thus, H. McManus in ref. 4 establishes a model of QED with nonlocal interaction, of which the action is 
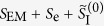
, where *S*_EM_, *S*_e_ and 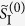 are given by (1), (2) and (7), respectively. H. McManus in ref. 4 proves that the theory is invariant under the gauge transformation (4), but now, the relation between the two functions *χ*(*x*) and *θ*(*x*) in (4) reads


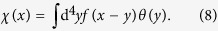


Specifically, by choosing 
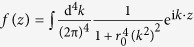
, where *r*_0_ is a constant, a theory of classical electrodynamics without singularities is obtained in ref. 4. However, M. Chretien and R. E. Peierls in ref. 5 point out that this theory is incapable of removing the divergence of the vacuum polarization in the corresponding quantum theory.

On the other hand, 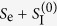, in which *S*_e_ and 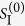 are given by (2) and (3), respectively, can be written in the form^8^


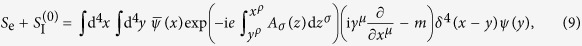


where the integral 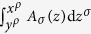 is taken over the straight line in space-time joining *x*^*ρ*^ and *y*^*ρ*^. By replacing *δ*^4^(*x* − *y*) in (9) by a scalar function *f*(*x* − *y*), which is independent of *ψ*(*x*) and *A*^*μ*^(*x*), M. Chretien and R. E. Peierls in ref. 5 establish a different model of nonlocal QED, of which the action is 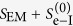, where *S*_EM_ is still given by (1), and


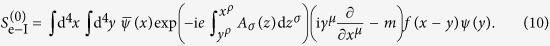


Although the form of this theory is far from that of conventional field theory, the theory is still invariant under the gauge transformation (4) and (5). For this theory, current conservation is no longer the normal form (6), and there still exist divergences in the theory^5^.

K. Scharnhorst in ref. 6 investigates a generalization of the above theory, of which the action is 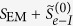, where *S*_EM_ is still given by (1), and


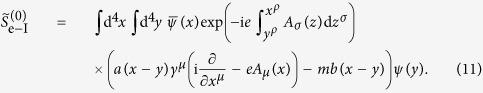


## Class of models of quantum electrodynamics with nonlocal interaction

A generalization of (7) is to replace *x*^*λ*^ and *y*^*λ*^ in the function *f*(*x*^*λ*^ − *y*^*λ*^) in (7) by two four-vector functions *U*^*λ*^(*x*) and *V*^*λ*^(*y*), respectively, 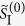 thus becomes


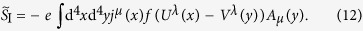


However, we can prove that the theory whose action is 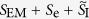, where *S*_EM_, *S*_e_ and 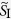 are given by (1), (2) and (12), respectively, breaks gauge invariance, no matter how the relation between the two functions *χ*(*x*) and *θ*(*x*) in (4) is chosen.

In order to restore gauge invariance, for the two functions *U*^*μ*^(*x*) and *V*^*μ*^(*y*), we introduce


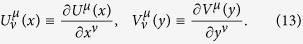


If we regard 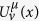 and 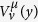 as two 4 × 4 matrices, then we ask that both the corresponding determinants *U*(*x*) and *V*(*y*) do not vanish:


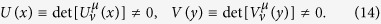


Hence, the corresponding inverse matrices 
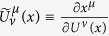
 and 
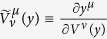
 exist and satisfy


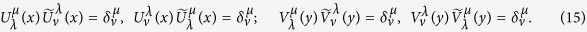


Taking advantage of the functions 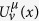, *V*(*y*) and 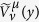 corresponding to *U*^*μ*^(*x*) and *V*^*μ*^(*y*), respectively, we construct an interaction action


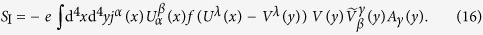


We ask that *f*(*z*) be independent of *ψ*(*x*) and *A*^*μ*^(*x*); *U*^*μ*^(*x*) and *V*^*μ*^(*y*) can be dependent on *ψ*(*x*) and *A*^*μ*^(*x*), but both of them are invariant under the transformation (4). Under these conditions, we now prove that the action *S*_EM_ + *S*_e_ + *S*_I_, where *S*_EM_, *S*_e_ and *S*_I_ are given by (1), (2) and (16), respectively, is invariant under the gauge transformation (4), but now, the relation between the two functions *χ*(*x*) and *θ*(*x*) in (4) reads


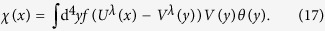


At first, it is easy to obtain that under the transformation (4), the sum of 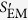 and 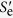, in which the field quantities are 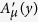 and *ψ*′(*x*), becomes


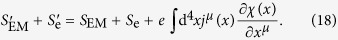


On the other hand, under the transformation (4), *j*′^*α*^(*x*) = *j*^*α*^(*x*) and 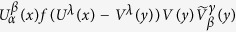 are invariant, because *f*(*z*) is independent of *ψ*(*x*) and *A*^*μ*^(*x*), and both *U*^*μ*^(*x*) and *V*^*μ*^(*y*) are invariant; therefore, under the transformation (4), 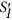, in which the field quantities are 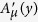 and *ψ*′(*x*), becomes


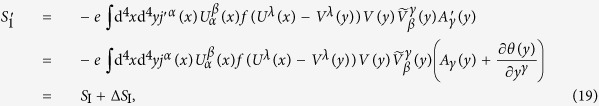


where *S*_I_ is expressed by (16), and


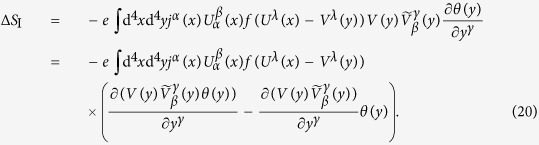


In the last section of this paper, we shall prove


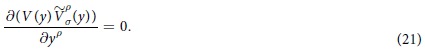


Thus, the last term in (20) vanishes; by an integration by parts with respect to *y*^*λ*^, Δ*S*_I_ becomes


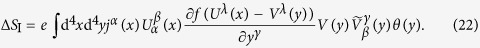


In the last section of this paper, we shall prove


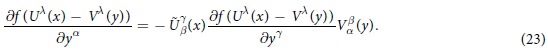


Substituting (23) into (22) and using some of the formulas in (15), Δ*S*_I_ becomes


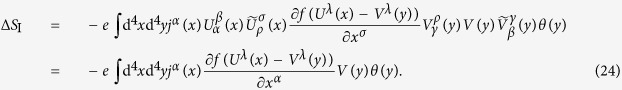


Combining (18) with (19), and using (24), we obtain


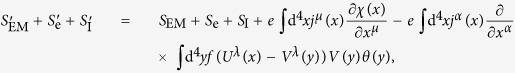


thus, if the function *χ*(*x*) is as introduced by (17), then we obtain 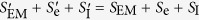; this means that the action *S*_EM_ + *S*_e_ + *S*_I_ is invariant under the gauge transformation (4) and (17).

It is obvious that the action *S*_EM_ + *S*_e_ + *S*_I_ has Lorentz invariance; therefore, *S*_EM_ + *S*_e_ + *S*_I_ is Lorentz and gauge invariant. However, if both *U*^*μ*^(*x*) and *V*^*μ*^(*y*) are independent of *ψ*(*x*) and *A*^*μ*^(*x*), then it is difficult to find two functions *U*^*μ*^(*x*) and *V*^*μ*^(*y*) such that *S*_EM_ + *S*_e_ + *S*_I_ has translation invariance. Contrarily, if *U*^*μ*^(*x*) and *V*^*μ*^(*y*) are dependent on *ψ*(*x*) or *A*^*μ*^(*x*), then it is easy to choose *U*^*μ*^(*x*) and *V*^*μ*^(*y*) such that *S*_EM_ + *S*_e_ + *S*_I_ has translation invariance.

For example, if we take


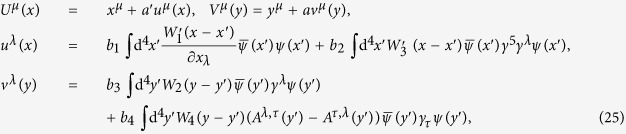


where *a*′, *a* and *b*_*k*_ are all constants (some constants can be zero), and 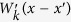 and *W*_*k*_(*y* − *y*′) are independent of *ψ*(*x*) and *A*^*μ*^(*x*), then it is obvious that *u*^*λ*^(*x*) and *v*^*λ*^(*y*), as well as *U*^*μ*^(*x*) and *V*^*μ*^(*y*), are invariant under the transformation (4), and both *U*^*μ*^(*x*) and *V*^*μ*^(*y*) satisfy (14). Besides these properties, according to translation invariance of the field quantities *ψ*(*x*) and *A*^*μ*^(*x*), and using


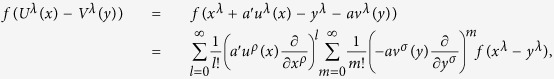


we can prove that *S*_EM_ + *S*_e_ + *S*_I_ has translation invariance.

Of course, besides (25), there are many choices for *U*^*μ*^(*x*) and *V*^*μ*^(*y*) such that *S*_EM_ + *S*_e_ + *S*_I_ has translation invariance.

On the other hand, in the appropriate limit, *S*_EM_ + *S*_e_ + *S*_I_ should reduce to the conventional local QED. This condition can be realized very easily; for example, 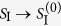 in the limit *a*′ → 0, *a* → 0 in (25) and *f*(*z*) → *δ*^4^(*z*).

Although the function *f*(*z*) in (7), (10) and (16) seems to be arbitrary, it must satisfy *f*(*z*) → *δ*^4^(*z*); hence, all different forms of *f*(*z*) have the same feature. On the other hand, the characteristics of *S*_EM_ + *S*_e_ + *S*_I_ vary with the forms of the two functions *U*^*μ*^(*x*) and *V*^*μ*^(*y*) in (16); therefore, *S*_EM_ + *S*_e_ + *S*_I_ represents a class of models rather than a single model.

We now take


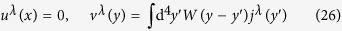


in (25) and *f*(*z*) = *δ*^4^(*z*) as an example to show some properties of the corresponding equations of motion of a charged particle and electromagnetic field. For this choice, (16) becomes


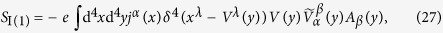


where *V*^*λ*^(*y*) is given by (25) and (26), i.e.,


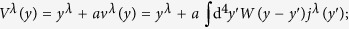


*V*(*y*) and 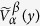 are given by (58) and (59), respectively, in the last section of this paper, and *v*^*λ*^(*y*) in (58) and (59) is given by (26).

From the action *S*_EM_ + *S*_e_ + *S*_I(1)_ and the action principle, we obtain the equations of motion of the electromagnetic field and charged particle:


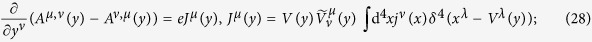



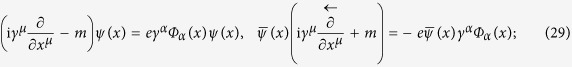



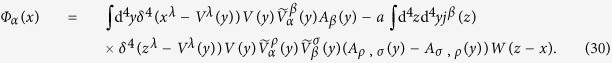


It is obvious that (28) is invariant under the transformation given in (4) and (17). By the same method as in the proof that the action *S*_EM_ + *S*_e_ + *S*_I_ is invariant under the gauge transformation (4) and (17), we can prove that (29) has the same gauge invariance.

It is easy to prove that the equation of motion (29) of a charged particle leads to the current conservation (6); on the other hand, as is well known, a very important and basic property of the equation of motion of the electromagnetic field 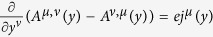 of the conventional local QED is that which leads to the current conservation (6) by the approach 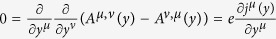. We prove that (28) has the same property.

From (28), we have


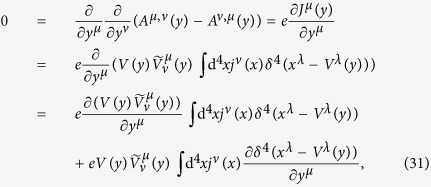


according to (21), the first term in the last expression of (31) vanishes; according to (23), we have 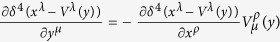. Thus (31) becomes


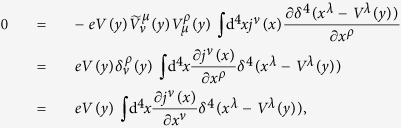


where we have used some of the formulas in (15) and performed integration by parts with respect to *x*^*λ*^. From the above result, we obtain the current conservation (6).

In the above discussions, *U*^*μ*^(*x*) and *V*^*μ*^(*y*) are mainly dependent on *ψ* and 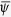; on the other hand, *U*^*μ*^(*x*) and *V*^*μ*^(*y*) can be dependent on *A*^*λ*^ alone. For example, if we take *u*^*λ*^(*x*) = 0 and 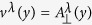 in (25) and *f*(*z*) = *δ*^4^(*z*), where 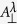 is the transverse four-vector corresponding to electromagnetic four-potential *A*^*μ*^, whose formal definition can be found in ref. 9,10, then (16) becomes


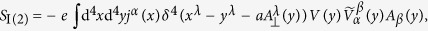


where *V*(*y*) and 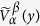 are still given by (58) and (59), respectively, in the last section of this paper, but now in (58) and (59), 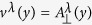.

For this case, from the action *S*_EM_ + *S*_e_ + *S*_I(2)_, we obtain the equations of motion of the charged particle and electromagnetic field:


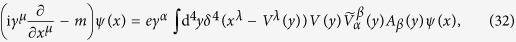



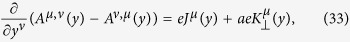


where *J*^*μ*^(*y*) is given by (28), 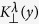 is the transverse four-vector corresponding to *K*^*μ*^(*y*), and


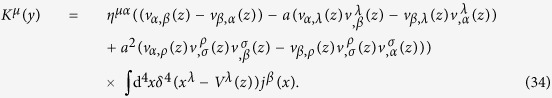


The action *S*_EM_ + *S*_e_ + *S*_I(2)_ and two equations of motion (32) and (33) are invariant under the gauge transformation (4) and (17), since the transverse four-vector 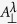 is invariant under the gauge transformation (4); besides, it is easy to prove that the equation of motion (32) of the charged particle leads to the current conservation (6), and, using the above method that proves that (28) leads to (6), we can prove that the equation of motion of the electromagnetic field (33) leads to (6).

Similar to (9), it is easy to prove that *S*_e_ + *S*_I_ can be written in the form


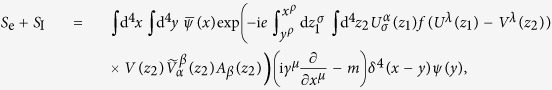


hence, similar to (10), by replacing *δ*^4^(*x* − *y*) in the above formula by a scalar function *g*(*x* − *y*) that is independent of *ψ*(*x*) and *A*^*μ*^(*x*), we can establish a class of models of nonlocal QED, of which the action is *S*_EM_ + *S*_e−I_, where *S*_EM_ is still given by (1), and


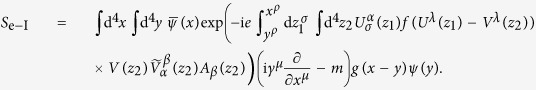


We can further extend the above action in terms of (11).

## Quantization of the models

From (28) and (29), or (32) and (33), we see that the free charged particle and free electromagnetic field still obey the local Dirac equation and the local Maxwell equation of free fields, respectively. The models thus belong to the so-called “theory with nonlocal interaction”. For this theory, so far, the unique method of quantization is to employ the formalism based on the Yang-Feldman equations^11^,^12^. Here, we list only the main steps of quantization of the models as follows. In the last step, we include brief discussions, since we shall employ the Lehmann-Symanzik-Zimmermann reduction formulas instead of the *S*-matrix.

The method of quantization works in the Heisenberg Picture; for simplicity, we employ the Coulomb gauge ∇ · ***A*** = 0 for electromagnetic four-potential *A*^*μ*^.

I     “in” and “out” fields and the Fock space.

The field operators *ψ*_in(out)_(*x*), 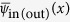 and 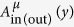 of the “in” and “out” fields satisfy the equations 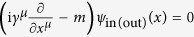, 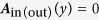, and ∇ · ***A***_in(out)_(*y*) = 0, respectively, for which the concrete expressions are given by (13.50) and (14.33) in ref. 7. All states of the corresponding Fock space are generated by repeatedly acting with the creation operators 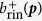, 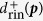 and 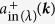 in the expressions of *ψ*_in(out)_(*x*), 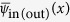 and 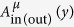 on the vacuum state 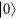.

II     The asymptotic conditions and the Yang-Feldman equations.

Using the idea of “weak convergence”^7^,^13^,^14^, the asymptotic conditions for the field operators *ψ*(*x*), 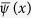 and ***A***(*y*) read:
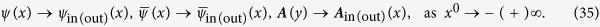


And, furthermore, considering the asymptotic conditions (35), from the equations of motion (30) and (28) we obtain the Yang-Feldman equations^7^:
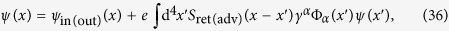

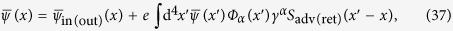

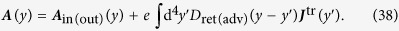


In (38), ***J***^tr^(*y*) is the transverse three-vector corresponding to *J*^*μ*^(*y*) introduced by (28). If the equations of motion of the charged particle and electromagnetic field are (32) and (33), respectively, then it is easy to write out the corresponding Yang-Feldman equations, similar to (36)~(38); besides, under the Coulomb gauge and the asymptotic conditions (35), the transverse four-vector 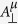 corresponding to *A*^*μ*^ reads
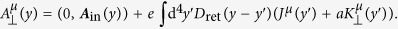


III     The Lehmann-Symanzik-Zimmermann (LSZ) reduction formulas

The last step of the method of quantization employing the formalism based on the Yang-Feldman equations is to introduce the *S*-matrix by


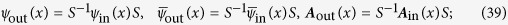


some examples of calculating the *S*-matrix in terms of (39) can be found in refs 15, 16.

However, generally speaking, for infinite-dimensional matrices *ψ*_in(out)_(*x*), 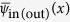 and ***A***_in(out)_(*y*), perhaps such a matrix *S* does not exist^7^,^13^; even if it exists, it may not be unitary^11^.

Conversely, for the theory with nonlocal interaction, we can prove that the LSZ reduction formulas^7^,^13^,^14^ still hold. Hence, we employ the LSZ reduction formulas instead of the *S*-matrix to calculate the transition amplitude. For example, for the case that the initial and final states are an electron, the corresponding transition amplitude is


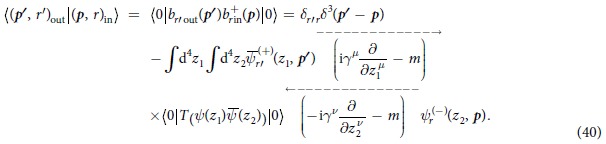


Substituting the field operators *ψ*(*x*) and 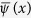 obtained from (36)~(38) by an iterative method into (40), we can calculate the transition amplitude 〈(***p*****′**, *r*′)_out_|(***p**, r*)_in_〉.

A question naturally arises from the above discussions: now that the transition amplitude is only an element of the *S*-matrix, if the *S*-matrix defined by (39) does not exist, or exists but is not unitary, then is the transition amplitude determined by the LSZ reduction formula correct?

Here, we only address this question briefly. Notice that there are in fact two types of *S*-matrices: one is defined based on operators, e.g., introduced by (39); the other is defined based on a state vector^7^,^13^,^14^ whose element, i.e., transition amplitude, can be determined by the LSZ reduction formula. The differences between the two types of *S*-matrices are subtle. For example, the asymptotic conditions (35) based on operators are in fact incorrect; the correct forms of the asymptotic conditions are based on the state vector^7^,^13^,^14^:


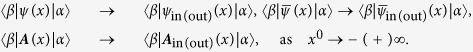


(The exact expressions of the asymptotic conditions can be found in ref. 14, e.g., the formulas 7(106a) and 7(106b) in ref. 14). And, thus, instead of (36)~(38), the correct forms of the Yang-Feldman equations should be^14^


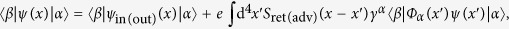


etc. Hence, regardless of whether the *S*-matrix based on operators exists, we can introduce the type of *S*-matrix based on the state vector, and, in addition, take advantage of the LSZ reduction formula to determine the element of this type of *S*-matrix, i.e., the transition amplitude.

We therefore obtain a complete quantum theory of the models of QED with nonlocal interaction, as presented in the above section.

However, although we employ the LSZ reduction formulas instead of the *S*-matrix, basic principles such as Lorentz and gauge invariance, unitarity and causality of the *S*-matrix must be satisfied, of course. We now ask that the elements of *S*-matrix, i.e., transition amplitude, satisfy these principles. And, furthermore, according to the LSZ reduction formulas, the question of whether the elements of the *S*-matrix satisfy these principles is reduced to investigating the properties of such Green functions as 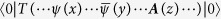.

For example, it is obvious that if such Green functions as 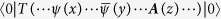 are Lorentz and gauge invariant, then the elements of the *S*-matrix are as well. And, the condition of unitarity corresponding to that of the *S*-matrix is now 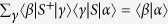, which also leads to the investigation of the properties of such Green functions as 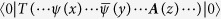.

As for the condition of causality of the *S*-matrix, following N. N. Bogoliubov and D. V. Shirkov^17^, we write the interaction action (16) in the form


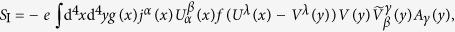


where 0 ≤ *g*(*x*) ≤ 1. When *g*(*x*) = 0, the interaction does not exist; when 0 < *g*(*x*) < 1, the interaction is joined partly; when *g*(*x*) = 1, the interaction is joined fully. Because the function *g*(*x*) appears in *S*_I_, it will appear in the equations of motion of the charged particle and electromagnetic field, and, additionally, in such Green functions as 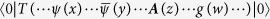 and the elements of the *S*-matrix 〈*β*|*S*(*g*)|*α*〉. Therefore, determining whether the elements of the *S*-matrix satisfy the condition of causality


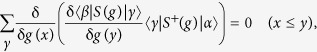


which corresponds to the condition of causality of the *S*-matrix given by ref. 17 (see the formula (17.30) in ref. 17), is still reduced to investigating the properties of such Green functions as 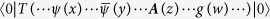.

On the other hand, experiences with some known theories show that the properties mentioned above of Green functions can be investigated only after the theories are researched deeply. For example, if we employ the Coulomb gauge in the conventional local QED, then Lorentz and gauge invariance of the elements of the *S*-matrix can be proved only after the rules of the Feynman diagrams of the theory are obtained (see ref. 7, Chapter 17). A different example is unitarity of the *S*-matrix of the theory of the non-Abelian gauge field: although as early as 1963, R. P. Feynman pointed out that one must add some additive terms in the theory of the non-Abelian gauge field to guarantee unitarity of the *S*-matrix under Feynman gauge^18^, and even though a general approach of adding additive terms was given by L. Fadeev and V. N. Popov^19^, the unitarity of the *S*-matrix of the theory of the non-Abelian gauge field can be proved only after Slavnov-Taylor identities are established.

What should we do when the models presented in this paper do not satisfy unitarity or (and) causality? A revelation from the theory of the non-Abelian gauge field is that, if the elements of the *S*-matrix of the models presented in this paper do not satisfy unitarity or (and) causality, then maybe we can add some appropriate additive terms to restore unitarity and causality, similarly to the addition of ghost fields to restore the unitarity of the *S*-matrix in the theory of the non-Abelian gauge field. These questions will be studied further.

## Calculation result of the vacuum polarization

Concrete calculation of the transition amplitude based on the LSZ reduction formula is lengthy and complex even for the conventional local QED. For example, for the conventional local QED, (correspondingly, *Φ*_*α*_ = *A*_*α*_ and *J*^*μ*^ = *j*^*μ*^ in (36)~(38)), in order to obtain the self energy of the electron and the vacuum polarization in terms of (40), we must calculate the field operators *ψ*(*x*) and 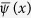 in terms of (36)~(38) by an iterative method up to second-order and third-order approximations, respectively. Of course, the vacuum polarization can be obtained from the self energy of the photon, but for this, we still must calculate the field operator ***A***(*x*) in terms of (36)~(38) by an iterative method up to second-order approximation. On the other hand, G. Källén in ref. 20 presents an approach to obtain the vacuum polarization for the conventional local QED: the approach is to calculate the linear response of the vacuum expectation value of current to an external electromagnetic field without using the LSZ reduction formula; one only needs to calculate the field operators *ψ*(*x*) and 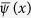 in terms of (36) and (37) by an iterative method up to first-order approximation. Here we follow this approach to calculate the vacuum polarization; of course, in (36) and (37), *Φ*_*α*_(*x*) is now given by (30).

The concrete calculation process is lengthy and complex; here we only present the main steps and results.

According to (36) and (37), up to first-order approximation, we have


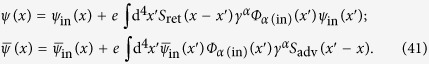


In (41), *Φ*_*α*(in)_(*x*) is given by (30), in which all field quantities are zero-order approximations, namely,


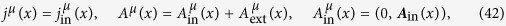


where 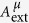 is an external electromagnetic field.

If we use


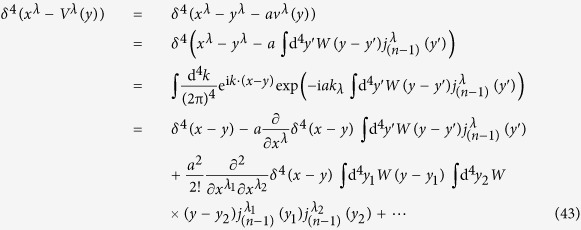


to deal with the factor *δ*^4^(*x*^*λ*^ − *V*^*λ*^(*y*)) in (30), then we should in principle calculate all terms in (43); in other words, we should calculate all orders of the parameter *a* in (43).

Based on (43), we obtain


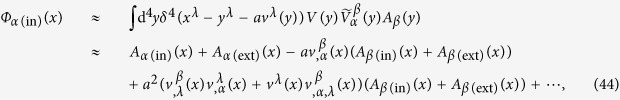


where we have used the expressions (58) and (59) of *V*(*y*) and 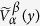 in the last section of this paper, respectively, and ignored all derivative terms of 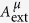, since what we calculate is the linear response of the vacuum expectation value of the current to 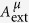.

Since the function *W*(*x* − *y*) in (26) is a (real) function but not a matrix, we can choose


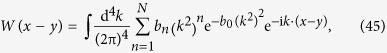


or by taking advantage of^20^


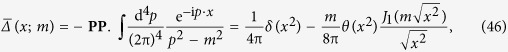


where *J*_1_(*z*) is a Bessel function, we choose


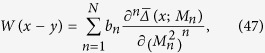


where all *b*_*n*_ and *M*_*n*_ are real constants.

Substituting (44) into (41), a problem of operator ordering of *ψ*_in_ and 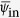 arises; we can prove that all operator orderings of *ψ*_in_ and 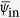 in (41) can be determined by the current conservation (6).

Based on the above discussions, we obtain


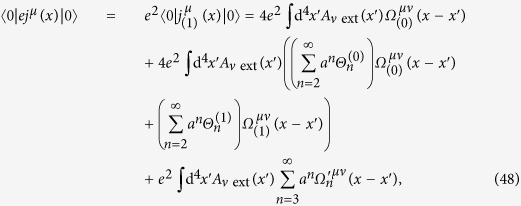


where all 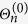 and 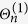 (*n* ≥ 2) are constants arising from (43) and (47),


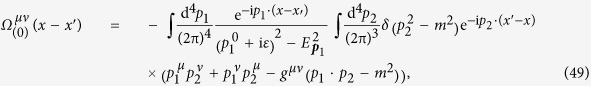



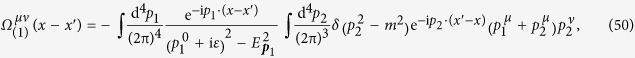


and 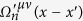 (*n* ≥ 3) in (48) are additional terms that cannot be written in the form of the second term in (48). Notice that no term is proportional to *a*^1^(=*a*) in (48), since the traces of the *γ*-matrices in this term vanish; this result is obtained by the software FeynCalc.

We can prove that the first term in (48) is exactly the same as the results (2.18) and (2.20) in ref. 21. J. Schwinger in ref. 21 writes 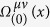, given by (49), in the form


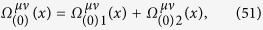



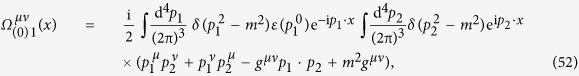



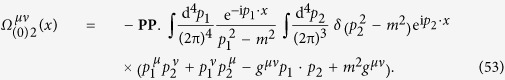


J. Schwinger in ref. 21 proves that if no real pair creation has occurred, then 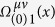 introduced by (52) vanishes; this conclusion is proved in the Interaction Picture in ref. 21, but here we work in the Heisenberg Picture, and, thus, have to use a different method to deal with 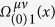.

The method we use is to calculate 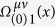 straightforwardly. This method can not only prove J. Schwinger’s conclusion in ref. 21, but also obtain a concrete result for 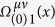 for the case that real pair creation has occurred.

The calculation result of 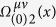 given by J. Schwinger in ref. 22 is


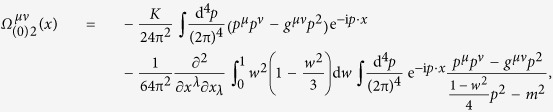


where


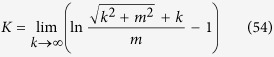


is logarithmically divergent.

We follow the above approach to deal with 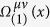 given by (50); for the case that no real pair creation has occurred, we finally obtain


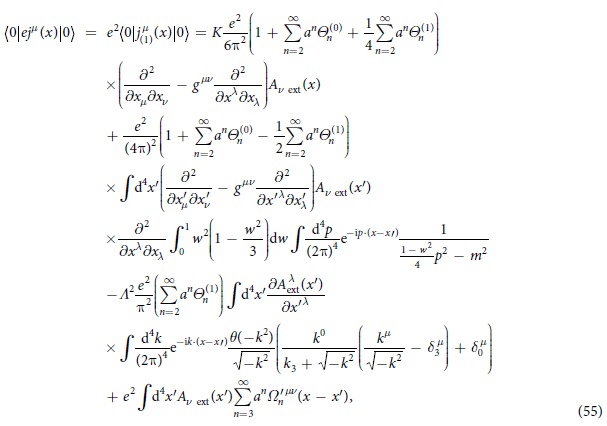


where *K* is given by (54), 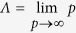, and the third term including *Λ* is a new divergent term arising from the new theory.

From (55), we see that

1. By choosing constants 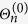 and 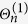 satisfying
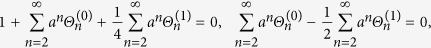
the first (divergent) term in (55) is eliminated and the second term in (55) presents the lowest nontrivial order of the vacuum polarization given by the formula (A.39) in ref. 21.
2. The third term (new divergent term) in (55) arising from the new theory can be eliminated by imposing Lorenz gauge 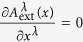 on external electromagnetic field 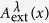.
3. We have not dealt with 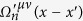 (*n* ≥ 3) in the last term in (55). It is possible that some terms arising from 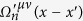 are similar to the first three terms in (55), and, thus, contribute to the first three terms in (55).
4. Generally speaking, since the new theory brings many new terms, it can be predicted that new divergent terms in the new theory presented in this paper will appear. Just as the method of higher covariant derivative regularization of gauge theories^23^,^24^,^25^ removes divergences in the original theory while at the same time bringing many new divergent terms, of course, all divergences are removed finally. We predict that the divergences brought by new terms arising from the new equations of motion (28) and (30) can be removed finally. For example, even if we don’t impose the Lorenz gauge on the external electromagnetic field, the third term (new divergent term) in (55) cannot be eliminated; however, it is possible that it can be eliminated by some terms arising from the last term in (55).
5. Our purpose is to establish a theory of QED without divergence, but there are still two divergent constants *K* and *Λ* in (55). For this result we emphasize that (I) both *K* and *Λ* arise from the calculation approach from (43) to (48); and (II) it is because of the structure and properties of the theory, but not renormalization procedure, that both *K* and *Λ* are eliminated. As for the calculation approach from (43) to (48), which not only leads to a complex computation process, but also causes some divergent integrals, e.g., (49) and (50), strictly speaking, the divergent integrals bring uncertainty, especially for the case of changing variables in the integral. Hence, seeking a different calculation approach for the models presented in this paper, especially for the treatment method of the *δ* - function given by (43), is necessary. Can we find a calculation approach such that there is no divergence in the calculation result? This question will be studied further.

Finally, we emphasize that the theory we employ in this section to calculate the vacuum polarization is only a special choice of the models presented in the section “A class of models of quantum electrodynamics with nonlocal interaction” of this paper. Concretely, the theory employed in this section is the result of defining the two functions *U*^*μ*^(*x*) and *V*^*μ*^(*y*) in (16) by (25) and (26), and, also of defining the function *W*(*y* − *y*′) in (26) by (45) or (47). On the other hand, the models presented in the section “A class of models of quantum electrodynamics with nonlocal interaction” of this paper provide a wide range of choices. Hence, maybe it is possible to establish a theory of QED without divergence by choosing appropriate functions *f*(*z*), *U*^*μ*^(*x*) and *V*^*μ*^(*y*) in the action (16).

## Proofs of (21) and (23)

Both (21) and (23) play important roles in the proofs of the gauge invariance of the model and the conclusion that the equation of motion of the electromagnetic field (28) leads to the current conservation (6). Eqs (21) and (23) can be proved by a basic function algorithm. Here we prove them by straightforward calculation. Some expressions obtained in the proof process are also used in the calculation of the vacuum polarization.

In order to prove (21), without loss generality, we assume


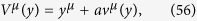


since the arbitrary vector function *V*^*μ*^(*y*) can always be written in such a form. According to (13),


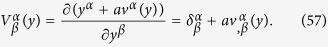


By a straight forward calculation, we obtain the determinant *V*(*y*) and the inverse matrix 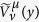 of 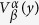 as follows.


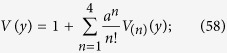



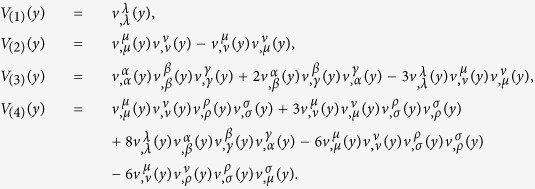



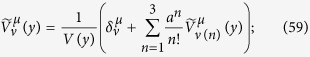



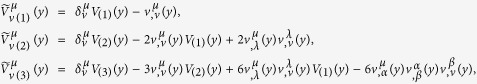


where *V*_(1)_(*y*), *V*_(2)_(*y*) and *V*_(3)_(*y*) are introduced by (58).

Therefore, 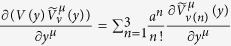, and according to the above expressions of 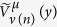, we can verify that 
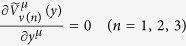
. Thus, (21) is proved.

In order to prove (23), based on the Fourier transform 
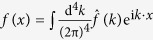
 of *f*(*x*), we have


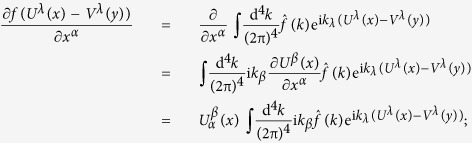


similarly,


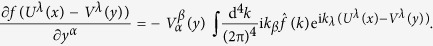


From the above two expressions and using some of the formulas in (15), we obtain


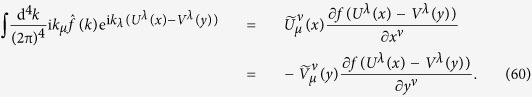


According to (60), each of 
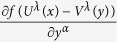
 and 
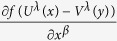
 can be transformed to the other; for example, from (60) we have


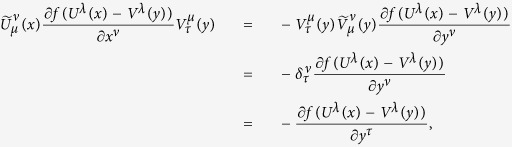


which is just (23).

When we use a basic function algorithm to prove (60), we do not need to take advantage of the form of the Fourier transform of *f*(*x*).

## Additional Information

**How to cite this article**: Mei, T. Class of gauge-invariant models of quantum electrodynamics with nonlocal interaction. *Sci. Rep.* **7**, 45386; doi: 10.1038/srep45386 (2017).

**Publisher's note:** Springer Nature remains neutral with regard to jurisdictional claims in published maps and institutional affiliations.
